# The roles of machine learning methods in limiting the spread of deadly diseases: A systematic review^☆^

**DOI:** 10.1016/j.heliyon.2021.e07371

**Published:** 2021-06-23

**Authors:** Rayner Alfred, Joe Henry Obit

**Affiliations:** Knowledge Technology Research Unit, Faculty of Computing and Informatics, Universiti Malaysia Sabah, 88400 Kota Kinabalu, Sabah, Malaysia

**Keywords:** Machine learning, Infectious disease, Disease outbreak, Prediction, Detection

## Abstract

Machine learning (ML) methods can be leveraged to prevent the spread of deadly infectious disease outbreak (e.g., COVID-19). This can be done by applying machine learning methods in predicting and detecting the deadly infectious disease. Most reviews did not discuss about the machine learning algorithms, datasets and performance measurements used for various applications in predicting and detecting the deadly infectious disease. In contrast, this paper outlines the literature review based on two major ways (e.g., prediction, detection) to limit the spread of deadly disease outbreaks. Hence, this study aims to investigate the state of the art, challenges and future works of leveraging ML methods to detect and predict deadly disease outbreaks according to two categories mentioned earlier. Specifically, this study provides a review on various approaches (e.g., individual and ensemble models), types of datasets, parameters or variables and performance measures used in the previous works. The literature review included all articles from journals and conference proceedings published from 2010 through 2020 in Scopus indexed databases using the search terms *Predicting Disease Outbreaks* and/or *Detecting Disease using Machine Learning*. The findings from this review focus on commonly used machine learning approaches, challenges and future works to limit the spread of deadly disease outbreaks through preventions and detections.

## 1 Introduction

The current global population of 7.8 billion (2020) persons is expected to reach 9.7 billion by 2050 [1]. Unfortunately, this population growth drives infectious disease rate upward [2]. There are many factors that contribute to disease emergences. These factors include climate change, globalization and urbanization, and most of these factors are to some extent caused by humans. Pathogens may be prone to emergence in themselves, and rapidly mutating viruses are more common among the emerging pathogens. Infectious disease occurs when a pathogen from a person can infect another person or an animal. It can cause harm on a macro scale such as the coronavirus COVID-19 and therefore can be considered as a major social problem. It not only harms individuals, but also causes harm on a macro scale and, therefore, is regarded as a social problem [3]. Thus, identification of high-risk areas for deadly infectious and non-infectious disease outbreaks is very importance so that prediction and detection of the deadly disease outbreaks can be conducted and responding to these deadly disease outbreaks can be made more effectively. Health agencies can leverage Machine Learning (ML) approaches in several ways to limit the spread of deadly infectious disease outbreak (e.g., COVID-19) [4], [5]. This can be done by applying machine learning algorithms in predicting and detecting the deadly infectious disease and also in responding to the deadly infectious disease. Most reviews focus on the application AI technology generally in healthcare and did not discuss about the algorithms, datasets and performance measurements that were used. In contrast, this paper outlines the literature review based on two major ways (e.g., prediction and detection) in controlling the spread of deadly disease outbreaks.

In predicting the disease outbreak [6], [7], [8], the machine learning algorithms can be used to learn datasets that consist of information about known viruses, animal populations, human demographics, biology and biodiversity information, available physical infrastructures, cultural/social practices around the world and also the geolocation of the diseases to predict any outbreaks. For instance, Malaria outbreak prediction can be performed using Support Vector Machine (SVM) and Artificial Neural Network (ANN) models that use Average monthly rainfall, Temperature, Humidity, Total number of positive cases, Total number of Plasmodium Falciparum (pF) cases and outbreak occur in binary values *Yes* or *No*, as the predictors and Root Mean Square Error (RMSE) and Receiver Operating Characteristic (ROC) are used to measure the performance of the models [6].

Public-health officials can also make use of the Geographic Information System (GIS) data and spatial analytic methods can be used to derive information or predictions with more proactive in taking steps to prevent future outbreaks [9]. Geographic information technology can be used to extract the spatial location of cases and explore the temporal and spatial changes of the disease epidemic and its spatial relationship with other objects stored in the GIS [7].

In order to produce effective detection methods, the machine learning methods can be embedded into an intelligent system in order to gauge or mine social media data for indications of any outliers related to unusual flu symptoms [10]. For instance, Chae *at al.* proposed a deep learning approach to predict infectious diseases. In their work, the parameters of deep learning algorithms are optimized and at the same time incorporating social media data for better detection results [11]. The parameters involved include variables such as the number of confirmed infectious disease diagnoses occurrence, the number of daily naver search, the number of Twitter mentioning the disease, the average temperature and humidity for all South Korea.

Live data related to emergency medical service and ambulance data can also be extracted and analysed for anomalies by using any machine learning algorithm for a better process and a more efficient and effective algorithm in detecting an abnormal disease event with much faster.

In responding to the infectious disease outbreaks, making a very quick informed decision is very critical in order to reduce the damages caused by the impact of the disease outbreaks after a disease event is identified [3], [8]. Machine learning methods can also learn integrated multi-sources data related to travel schedule, population, logistics and epidemiology data in order to predict the disease's location and rate of spreading. For medical doctors, machine learning methods can be used to improve the application of current treatment and accelerate the time it takes to develop new treatments. For instance, they may use deep learning algorithms to model large data sets in order to learn any medical data captured by the hospitals. For example, data from clinical tests of coronavirus patients can serve as input for machine learning models so doctors can make faster diagnoses.

The aim of this study is to investigate the state of the art, challenges and future works of leveraging machine learning methods to control the spread of deadly disease outbreaks according to two categories mentioned earlier. This study provides a review on various approaches, types of datasets, types of parameters or variables, individual models, ensemble models, performance measures and approaches used in the previous works. The literature review included all articles from journals and conference proceedings published from 2010 through 2020 in Scopus indexed databases using the search terms *Predicting Disease Outbreaks* and/or *Detecting Disease using Machine Learning*. We categorized all articles and reports based on global health security issues addressed - i.e., whether it depicted prediction or detection strategies. The findings from this review focus on commonly used machine learning approaches, challenges and future works in controlling the spread of deadly disease outbreaks through preventions and detections.

## 2 Method

The aim of this Systematic Literature Review (SLR) [12] is to identify, evaluate and interpret all available research relevant to the application of machine learning approaches in limiting the spread of deadly disease outbreaks.

Five primary stages are identified to be included in this SLR. They are called Identification of Preliminary Requirement (IPR), Contents Retrieval (CR), Contents Evaluation (CE), Contents Summarization (CS) and Review Findings Reporting (RFR).

In the IPR stage, it involves activities to determine the requirements for a systematic review and it also serves to eliminate the possibility of researcher biases in reviewing all the papers by determining the appropriate review protocol. The objective of the review protocol is to ensure that the process of reviewing can be conducted unbiasedly. The most critical elements of the proposed review protocol in this work include all the outlined research questions, the process of searching relevant studies, inclusion and exclusion criteria, determining the quality assessment, knowledge extraction and data synthesis which details are explained in the next section.

The contents retrieval stage consists of formulating research questions that focus on the machine learning approaches that are leveraged to limit the spread of disease outbreaks and finally establishing the appropriate search process in order to conduct the search activities.

**Figure 1:**
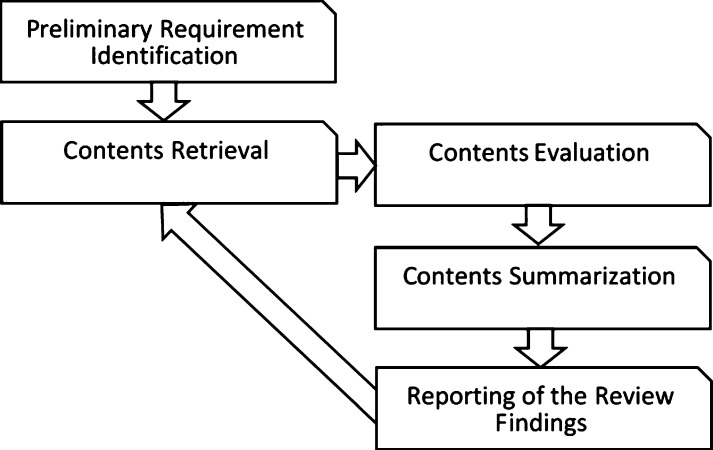
Five primary stages of the systematic literature review.

The contents evaluation stage involves the following steps: formulating the predefined selection criteria with the purpose of selecting relevant and assessing the quality of these studies based on the predefined quality assessment procedure outlined in this work. The contents summarization stage will then extract information obtained from the studies by performing data synthesis and to summarise the results. The final reporting of the review findings stage is presenting the findings and concluding this review with some future works derived from this review. All these processes are illustrated in Fig. 1 in which new information can be integrated into the report in the future.

## 3 Contents retrieval

### 3.1 Formulating research questions

The research questions (RQs) were formulated to define the scopes of the research according to three viewpoints; *population*, *intervention* and *outcomes* [12]. The *population* viewpoint covers the areas or roles (e.g., prediction, detection and responses) affected by the intervention. The populations might be any of the following: The roles of specific machine learning method or the types of machine learning models and its application area. Then, the *intervention* viewpoint covers machine learning approaches that address specific issues, for example, machine learning approaches to perform specific tasks such as prediction of disease outbreak, detection of disease outbreak and responses to disease outbreak. Finally, the *outcomes* viewpoint should relate to factors of importance to practitioners such as improved prediction, reduced diagnosis costs for certain diseases, and reduced time to perform the detection of deadly disease outbreak. All relevant outcomes should be specified. For example, in some cases, we require interventions that improve some aspect of disease outbreak prediction without affecting another, e.g., improved reliability with no increase in cost. The primary objective of this SLR is to collect and analyse appropriate evidences to answer the outlined RQs. Our motivation is to answer a set of seven RQs to obtain insights into significant aspects of our research direction, including advancing our knowledge of the roles of ML technologies in limiting the spread of deadly disease outbreak and identifying the limitations of research so as to define further research directions. The RQs and their motivation are documented in Table 1.

**Table 1:** Research question.

ID	Research Question
RQ1	What are the roles of machine learning models in limiting the spread of deadly diseases outbreak?
RQ2	What disease datasets in the literature have been used to build the models?
RQ3	What type of parameters or variables have been used?
RQ4	What type of problems are addressed using these machine learning models?
RQ5	What are the individual models used?
RQ5.1	What are the best performing individual models?
RQ6	What are the evaluation measures and approaches used to assess the performance of the machine learning models?
RQ7	What type of ensemble models are used in the machine learning models?
RQ7.1	Do the ensemble models outperform the individual models?

### 3.2 Search process

The search process is conducted, and it must ensure that all the predefined research questions can be taken into consideration and thus this search process involves identifying the appropriate digital libraries, choosing the interval time of the published articles and defining search keywords.

Five most popular and largest computer science online digital libraries and a Medline digital library that publish peer-reviewed articles will be explored and these digital libraries are listed in Table 2.

**Table 2:** Online digital libraries.

No	Online Digital Libraries	Websites
1	Elsevier	https://www.sciencedirect.com/
2	Springer	https://link.springer.com/
3	IEEE eXplore	https://ieeexplore.ieee.org/
4	ACM Digital Library	https://dl.acm.org/
5	Wiley online library	https://onlinelibrary.wiley.com/
6	Medline (life sciences and biomedicine)	https://www.nlm.nih.gov/bsd/medline.html

Furthermore, several independent relevant journals and conference proceedings in the artificial intelligence field were explored which are presented in Table 4. The search was limited to articles published in the interval from 2010 to 2020. We restricted the search in this time interval since machine learning has been extensively used to be applied to problems related to diseases outbreak in 2010s.

We created a list of search strings by integrating appropriate synonyms and alternative terms with the Boolean operator (AND has the effect of narrowing and limiting the search, while OR serves to broaden and expand the search).

The following search terms were formulated in this SLR: (artificial intelligence OR disease outbreak), (artificial intelligence AND disease outbreak), (machine learning OR disease outbreak), (machine learning AND disease outbreak), (deep learning OR disease outbreak), (deep learning AND disease outbreak), (prediction OR disease outbreak), (prediction AND disease outbreak), (detection OR disease outbreak), (detection AND disease outbreak).

Since *deep* learning algorithm is one of the *machine learning* algorithms and *machine learning* is a subset of *artificial intelligence*, we decided to use these terms in this review. Thus, this paper focuses on a systematic summarisation of artificial intelligence techniques that include machine learning techniques and deep learning techniques used in predicting, detecting and responding the deadly disease outbreaks. The candidate studies were selected if they meet our criteria outlined in *Content Evaluation* section.

## 4 Contents evaluation

In the content evaluation phase, several criteria were carefully formulated in order to ensure that appropriate studies are selected. Table 3 shows the assessment criteria outlined in this work. Then, all retrieved studies were examined carefully. This quality assessment was performed according to the quality checklist proposed by Kitchenham [12]. The main objective of the assessment is to evaluate and select relevant studies that can be used to answer all the predefined research questions outlined in Table 1.

**Table 3:** Quality Assessment Question.

ID	Ten Assessment Questions
AQ1	Does the study define a main research objective or problem related to the spread of deadly diseases outbreak (e.g., prediction, detection, responses)?
AQ2	Does the study specify the relevant disease datasets used?
AQ3	Does the study specify the availability of these datasets (e.g. public datasets, private datasets)?
AQ4	Does the study define the parameters or variables used or learnt by the machine learning algorithms?
AQ5	Does the study define the type of parameters used or learnt by the machine learning algorithms?
AQ6	Does the study specify the type of machine learning models used (e.g. classification, regression, clustering) in solving the problem?
AQ7	Does the study specify the individual models explicitly (e.g., neural network, linear regression)?
AQ8	Does the study specify the evaluation measures (e.g., Accuracy, Precision, Recall, F-Measure, ROC) used to assess the performance of the proposed machine learning approach?
AQ9	Does the study specify the evaluation approaches (e.g., cross-validation, holdout) used to assess the performance of the proposed machine learning approach?
AQ10	Does the study specify the ensemble models (e.g., bagging, boosting) used and compare the performance with individual models?

The total scoring of the quality assessment by applying all the questions for a particular study, $S_{j}$, can be measured using the following formula:

(1) $$Score(S_{j})=\frac{1}{\left|AQ\right|}\sum_{i=1}^{\left|AQ\right|}AQ_{i,j}$$

where $\left|AQ_{i,j}\right|$ is the number of questions applied (e.g., which is 10 for AQ1 - AQ10), $AQ_{i,j}$ is the score for individual assessment question, *i*, for study *j*, in which the value of score is 1 if the answer is YES, 0.5 is the answer is partly and 0 if the answer is NO. Then, the paper is ranked according to the score computed in Eq. (1), as *Excellent* (0.85 $\leq Score(S_{j})\leq$ 1.00), *Good* (0.65 $\leq Score(S_{j})<$ 0.85), *Fair* (0.50 $\leq Score(S_{j})<$ 0.65) and *Poor* (0.00 $\leq Score(S_{j})<$ 0.50). Based on the above quality assessment criteria, we only consider studies that are ranked *Excellent* and *Good* only.

The number of studies retrieved, screened, reviewed and the average score of assessments for each study reviewed are summarized in Table 4 and the total of studies selected for each year are tabulated in Table 5.

**Table 4:** Number of studies screened and reviewed.

No	Online Digital Libraries	Retrieved	Screened	Reviewed	Average Score	Quality
1	Elsevier	987	54	18	0.889	Excellent
2	Springer	559	46	6	0.817	Good
3	IEEE eXplore	456	15	7	0.771	Good
4	ACM	380	13	7	0.814	Good
5	Wiley	28	8	1	0.800	Good
6	Medline (PubMed)	158	25	8	0.825	Good
			**Total**	**47**	0.838

**Table 5:** Number of studies reviewed based on year (2010 - 2020).

	2010 - 2015	2016	2017	2018	2019	2020
Studies	7	3	2	9	19	7

Based on the proposed assessment, forty-seven studies have been selected to be reviewed for this SLR. The search yielded 47 articles and publicly available reports from the computer science and the pubmed online digital library. Based on Table 5, majority of the papers reviewed are obtained from the publications between the year 2018 and 2020.

## 5 Contents summarization

Both quantitative and qualitative data were extracted from the selected studies that address issues related to the outlined research questions and the results are presented in the form of tables.

### 5.1 Roles of machine learning models

Disease outbreaks prediction and detection contributes to the improvement of the surveillance systems. Based on the type of problems addressed, most of the task of *predicting* the disease outbreaks or modelling the disease frequencies using regression methods. On the other hand, most of the classification problems solved by machine learning models are related to the task of *detecting* disease outbreaks. Table 6 tabulates all the studies related to prediction and detection of disease outbreaks.

**Table 6:** Type of Machine Learning Problems and Related Studies.

Problems	Roles	Related Studies
Regression	Predict disease outbreaks	[13], [14], [15], [16], [17], [18], [19], [20], [21], [22], [23], [24], [25], [26], [27], [28], [29], [30], [31], [32], [33], [34], [35], [36], [37], [38], [39], [40], [41], [42]
Classification	Detect disease outbreaks	[22], [43], [44], [45], [46], [47], [48], [49], [50], [51], [52], [53], [54], [55], [56], [57], [58], [59]

### 5.2 Types of datasets and parameters used

Table 7, Table 8 summarize both the structured and unstructured data based on the type of datasets used and also number of studies conducted for predicting and detecting the spread of disease outbreaks in this work. Based on these findings obtained from all the studies, there are six sets of structured datasets and three sets of unstructured datasets identified as the most commonly used in predicting and detecting the spread of disease outbreaks.

**Table 7:** Structured Data: Datasets and Parameters Used.

Databases (Frequency)	Features
Epidemiology Data (18)	Number of Disease Outbreak Incidences, Signs and Symptoms of Diseases, Treatment Information, Seasonal Information
Spatial Data (4)	GPS Coordinates, Topology, Distance, Area
Remotely Sensed Data (2)	Normalized Difference Vegetation Index, Normalized Difference Water Index, Land Surface Temperature
Meteorological Data (24)	Temperature, Humidity, Precipitation, Air Pressure, Solar Radiation, Wind Speed
Physiological Data (3)	Blood Pressure, Cholesterol, Obesity, Heart Rate, Risk Factor (e.g., Smoking)
Demographic Data (6)	Age, Gender, Race, Ethnicity, Marital Status, Income, Education, Occupation, Employment

**Table 8:** Unstructured Data: Datasets and Parameters Used.

Databases (Frequency)	Features
Social Media Data (12)	Posted Text, Post Time, Post Date, Post Geo-Location, Number of Comments, Number of Likes
Search Keywords (9)	Keywords Searched, Keywords Volumes, Keywords Trends
News Articles (1)	Original News Texts, News Published Date, Symptoms Detected

The structured databases include the *Epidemiology* Data, *Spatial* Data, *Remotely Sensed* Data, *Meteorological* Data, *Physiological* Data and finally *Demographic* Data. *Epidemiology* is a systematic study and analysis of the distribution, patterns and determinants of health and disease conditions in a particular predefined population. The three most used epidemiology parameters in this review include the number of disease outbreak incidences, signs and symptoms of diseases, treatment information and seasonal information. *Spatial* data, also known as geospatial data, is information about a physical object that can be represented by numerical values in a geographic coordinate system [60]. Other information includes digital elevations, distance and area. *Remotely Sensed* Data are derived from the remote sensing activities. Remote sensing is the process of detecting and monitoring the physical characteristics of an area by measuring its reflected and emitted radiation at a distance (typically from satellite or aircraft) [61]. Three parameters that are commonly included in the prediction that include Normalized Difference Vegetation Index (NDVI), Normalized Difference Water Index (NDWI) and Land Surface Temperature (LST). *Meteorological* data includes temperature, humidity, precipitation (Rain or Snow), air pressure, solar radiation and wind speed. They were collected regionally by surface and upper air meteorological stations [62]. *Physiological* data represents physiological properties including blood pressure, cholesterol, obesity, heart Rate and many others variables [63]. Finally, *demographic* data is statistical data collected about the characteristics of the population, e.g. age, gender, race, ethnicity, marital status, income, education and occupation for example.

Next, unstructured databases are typically large collections of files that are not stored in a structured database format. In this work, three types of unstructured datasets are found to be useful that include *Social Media* Data, *Search Keywords* and *News Articles*. Social media data (or social data for short) refers to all of the raw insights and information collected from individuals social media activity [64]. This social data includes posted texts, post time, post date, post location, number of comments and number of likes. *Keyword* research provides you with specific search data that can help you answer questions such as what are people searching for, how many people are searching for it and in what format do they want that information [65]. In this review, search keyword is one of the parameters used to predict the occurrence of disease outbreaks, and the parameters collected include the keywords searched, keywords volumes and keywords trends. Finally, *news articles'* parameters that are commonly used include the original news texts, news published date and symptoms detected in the news [66].

### 5.3 Type of problems addressed and individual machine learning models

Table 9 tabulates and summarizes the regression problems and all the individual machine learning models applied to achieve the objectives of each study. On the other hand, Table 10 tabulates and summarizes the classification problems and all the relevant individual machine learning models applied to solve these classification problems. The best models and their performances for each study are also tabulated in these tables. The details of the findings are discussed in Section 6.3. Based on the results shown in Table 9, for time-series data, ARIMA and LSTM are the most common machine learning algorithms used to perform the prediction [23], [25], [28], [31], [32], [33], [38], [39]. On the other hand, the family of ANN and *k*NN algorithms are widely used in solving the classification tasks [44], [46], [48], [49], [51], [55], [59].

**Table 9:** Regression: Types of Machine Learning approaches and Individual Models Used.

Study	Objectives	Models Applied	Best Model
[13]	Predicting the number of new outbreaks of diseases	ARMA(1,1), ARMA(1,0), ARMA(0,1)	ARMA(0, 1) (MAE = 1.257)
[14]	Incidence prediction of communicable diseases using remote sensing	BPNN	BPNN^⁎^ (MSE = 0.100)
[15]	Predicting dengue outbreak	HNN, ANN, NLR	HNN^⁎^ (MSE = 0.239)
[16]	Prediction of province-level outbreaks of foot-and-mouth disease	ZI	ZI
[17]	Forecasting influenza like illness	ARIMA, LASSO, LSTM, FNN, MARS	LSTM^⁎^ (MAPE = 0.320)
[18]	Antibiotic resistance outbreaks prediction	GPR, SVM, *k*NN, RF, LR, MLP	SVM (MAE = 0.100)
[19]	Forecasting the endemic infectious diseases	LASSO	LASSO (MAPE = 0.404)
[20]	Modeling Dengue vector population using remotely sensed data and machine learning	LR, RR, SVR, MLP, DTR, *k*NNR	MLP, *k*NNR (MSE = 0.494)
[22]	Predicting influenza outbreaks	ARIMA, SVM, RF, ANN	ANN^⁎^ (MAE = 0.119)
[23]	Predict infectious diseases	XGBoost, LSTM, RR, ARIMA	LSTM^⁎^ (MAPE = 0.099)
[25]	Prediction of Malaria disease outbreak	ARIMA, SARIMA, BPNN, LSTM	LSTM^⁎^ (RMSE = 0.072)
[26]	Time Series Analysis of Dengue Fever	SARIMA	SARIMA(1,2,2) (MAPE = 0.050)
[27]	Prediction of avian influenza H5N1 outbreaks	ARIMA, RF	RF (MSE = 0.248)
[28]	Predicting new and urgent trends in epidemiological data	RNN, LSTM	LSTM^⁎^ (RMSE = 0.140)
[29]	Predicting the spread of influenza epidemics by analyzing twitter messages	ARX, ARMAX, NARX, DeepMLP, CNN	CNN^⁎^ (MAE 0.250)
[30]	Predicting of Dengue outbreaks	*k*NN	*k*NN (RMSE = 0.089)
[31]	Influenza Trends Prediction	LSTM	LSTM^⁎^ (RMSE = 0.015)
[32]	Forecast of Dengue Cases in China	LSTM-TL, LSTMs, BPNN, GAM, SVR, GBM	LSTM-TL^⁎^ (RMSE = 0.322)
[33]	Predicting Infectious Disease in Korea	OLS, ARIMA, NN, LSTM	LSTM^⁎^ (RMSE = 0.179)
[38]	Forecasting Hepatitis incidence	ARIMA, RNN, ARIMA + RNN	ARIMA + RNN^⁎^ (MAPE = 0.045)
[39]	Prediction of Haemorrhagic fever with renal syndrome in China	ARIMA, RNN, ARIMA + RNN	ARIMA + RNN^⁎^ (MAPE = 0.178)
[40]	Forecasting dengue incidence in Guadeloupe, French West Indies	SARIMA	SARIMA (RMSE = 0.850)
[41]	Dengue prediction model based on climate	SARIMA	SARIMA (MSE = 0.839)
[42]	Forecasting incidence of hand, foot & mouth disease	ARIMA, BPNN	BPNN^⁎^ (MAPE = 0.200)

**Models:** Exogenous Inputs (ARX), Autoregressive Moving Average with Exogenous Inputs (ARMAX), Auto Regressive Integrated Moving Average (ARIMA), Autoregressive Moving Average (ARMA), Artificial Neural Network (ANN), Back Propagation Neural Network (BPNN), Convolutional Neural Network (CNN), Decision Tree Regression (DTR), Feedforward Neural Network (FNN), Gradient Boosting Machine (GBM), Gaussian Process Regression (GPR), Hybrid Neural Network (HNN), *k*-Nearest Neighbour (*k*-NN), *k*-Nearest Neighbour Regression (*k*-NNR), Least Absolute Shrinkage and Selection Operators (LASSO), Linear Regression (LR), Long Short Term Memory (LSTM), Multilayer Perceptron (MLP), Multivariate Adaptive Regression Splines (MARS), Nonlinear Autoregressive Exogenous (NARX), Non-Linear Regression (NLR), Random Forest (RF), Recurrent Neural Network (RNN), Ridge Regression (RR), Seasonal Autoregressive Integrated Moving Average (SARIMA), Support Vector Machine (SVM), Support Vector Regression (SVR), Zero-Inflated (ZI). **Note:**^⁎^Belongs to Neural Network family.

**Table 10:** Classification: Types of Machine Learning approaches and Individual Models Used.

Study	Objectives	Models Applied	Best Model
[22]	Predicting influenza outbreaks in Iran	SVM, RF, ANN	SVM (MAE = 0.132)
[43]	Detecting Disease Outbreaks among Physiological Variables	FL	FL (*F*_1_ Score = 0.820)
[44]	Predicting outbreak of hand-foot-mouth diseases	RR, *k*-NN, RF, LSTM	LSTM^⁎^ (ROC = 0.841)
[45]	Predicting death and cardiovascular diseases in dialysis patients.	LR, *k*-NN, CART, NB, SVC-RBF	SVC-RBF (ACC = 0.953)
[46]	Event detection and Situational Awareness of disease outbreaks	NB, SVM, LSTM	LSTM^⁎^ (*F*_1_ Score = 0.939)
[47]	Modelling disease outbreak events	CRF	CRF (*F*_1_ Score = 0.885)
[48]	Infection detection using physiological and social data in social environments	*k*NN	*k*NN (ROC = 0.798)
[49]	Detection and prevention of mosquito-borne diseases	NB, RDT, J48, F*k*NN	F*k*NN (ACC = 0.959)
[51]	Detecting the occurrence of Zika	BPNN, GBM, RF	BPNN^⁎^ (ROC = 0.966)
[53]	Influenza Detection and Surveillance	NB, ME, DLM	NB (ACC = 0.700)
[54]	Detection on Dengue Diseases	MAA	MAA (ACC = 0.750)
[55]	Detection of Meningitis Outbreaks in Nigeria	RF, ANN, *k*NN, LR, SVM	NN^⁎^ (ACC = 0.951)
[56]	Detecting global African swine fever outbreaks	RF	RF (ACC = 0.847)
[59]	Detecting disease epidemics using a symptom-based approach	M*k*NN	M*k*NN

**Models:** Artificial Neural Network (ANN), Back Propagation Neural Network (BPNN), Dynamic Language Model (DLM), Fuzzy k-Nearest Neighbor (F*k*NN), Fuzzy Logic (FL), Gradient Boosting Machine (GBM), Long Short Term Memory (LSTM), Classification Decision Tree (CART), Conditional Random Field (CRF), J48 classifier (J48), Linear Regression (LR), *k*-Nearest Neighbour (*k*-NN), Random Forest (RF), Maximum Entropy (ME), Modified Apriori Algorithm (MAA), Modified *k*-Nearest Neighbor (M*k*NN), Naive Bayes (NB), Random Decision Tree (RDT), Ridge Regression (RR), Support Vector Classifier RBF kernel (SVC-RBF), Support Vector Machine (SVM). **Note:**^⁎^Belongs to Neural Network family.

### 5.4 Assessment measures and methods

Various evaluation measures have been used in assessing the performance of the machine learning algorithms used to predict or detect disease outbreaks. These types of evaluation measures depend on the problem type: regression or classification. For instance, the Mean Absolute Error (MAE) [13], [18], [22], Mean Absolute Percentage Error (MAPE) [17], [19], [23], [26], [38], [39], [42], Root-Mean-Square Error (RMSE) [25], [28], [30], [31], [32], [33], [40] and Mean Squared Error (MSE) [15], [20], [20], [27], [41] evaluation measures are used to solve the regression problems and Accuracy [22], [49], [53], [54], [55], [56], F_1_ Score [43], [46], [47], AUC-ROC [44], [48], [51] evaluation measures are used to solve the classification problems [67]. The performance comparison of various approaches and metrics are discussed in more detail in Section 6.4.

### 5.5 Ensemble methods

Table 11, Table 12 outline the proposed ensemble approach to predict and detect disease outcomes and also summarize the evaluation approaches and measures used for ensemble learning.

**Table 11:** Ensemble Methods Used for Regression Problems.

Study	Objectives	Models Applied	Best Model
[21]	Forecasting influenza activity	SAAIM, LSTM, LASSO	SAAIM (MAPE = 0.104)
[24]	Predicting Influenza-like-illness (ILI) using multiple open data sources	AR, VAR, GPR, RNN, RNN-CNN, CNN-RNN-ResNet	CNN-RNN-ResNet (RMSE = 0.259)
[25]	Prediction of Malaria disease outbreak	ARIMA, SARIMA, BPNN, LSTM, ARIMA+SARIMA+BPNN+LSTM	ARIMA + SARIMA + BPNN + LSTM (RMSE = 0.068)
[34]	Prediction of dengue outbreak	EPRA, LASSO, RR, ENet	EPRA (MAE - 1.069)
[35]	Forecasting Ebola disease epidemic	GGM, GLM, GGM+GLM	GGM+GLM (RMSE = 0.374)
[36]	Forecasting respiratory syncytial virus outbreaks	Superensemble	Superensemble (MAE = 0.1011)
[37]	Forecasting seasonal influenza epidemic	XGBoost, LASSO, SAAIM	SAAIM (RMSE = 0.374)

**Models:** Autoregression (AR), Auto Regressive Integrated Moving Average (ARIMA), Back Propagation Neural Network (BPNN), Convolutional Neural Network (CNN), Elastic Net (ENet), Ensemble Penalized Regression Algorithm (EPRA), Generalized-Growth Model (GGM), Generalized Logistic Model (GLM), Long Short Term Memory (LSTM), Residual Neural Network (ResNet), Seasonal Autoregressive Integrated Moving Average (SARIMA), SARIMA + XGBoost (SAAIM), Least Absolute Shrinkage and Selection Operators (LASSO), VAR, GPR, Recurrent Neural Network (RNN), Ridge Regression (RR).

**Table 12:** Ensemble Methods Used for Classification Problems.

Study	Objectives	Models Applied	Best Model
[50]	Detecting and Classifying diseases	RKRE, SKRE, KG_ResNet	RKRE (ACC = 0.886)
[57]	Predicting Disease Risk	DPMM, COOC, CBC, eDPMM, eCOOC, eCBC	eCBC (ACC= 0.765)
[58]	Classification of risk areas using am ensembled bootstrap-aggregated	Ensemble DTs with bootstrap aggregating	eDT (ROC = 0.91)

**Models:** ResNet, Residual Neural Network (ResNet), ResNet + KG_ResNet (RKRE), Knowledge Graph + Residual Neural (KG_ResNet), SVM + KG_ResNet (SKRE), Dirichlet Process Mixture Mode (DPMM), DPMM trained on disease occurrence (COOC), Co-occurrence Based Clustering (CBC), Ensemble Dirichlet Process Mixture Mode (eDPMM), Ensemble DPMM trained on disease occurrence (eCOOC), Ensemble Co-occurrence Based Clustering (eCBC), Ensemble Decition Tree (eDT).

Ensemble model, that integrates multiple weak classifiers, tends to perform better than a single classifier. Table 11, Table 12 showed that combining several strong classifiers also improved the regression and classification results. There is a need to explore further the capability of ensemble models or hybrid models based on deep learning methods using multi-source data, as these have been shown to improve the performance of the base model. Section 6.5 discusses in detail about the findings obtained from this review related to the performance of ensemble methods in detecting and predicting disease outbreaks.

## 6 Reporting of review findings

In the reporting of review findings, the summary of findings was obtained from the selected studies based on the outlined research questions.

### 6.1 Roles of machine learning models

This section summarizes and discusses the findings in relation to the **RQ1**: What are the roles of machine learning models in limiting the spread of deadly diseases outbreak? The roles of machine learning models can be categorized into regression and classification problems.

#### 6.1.1 Regression problems for predicting disease outbreaks

Regression problems are commonly addressed in the task of predicting or modelling the disease frequencies as shown in Table 6. For instance, Li and Luan showed how ARMA model is applied to predict the number of new outbreaks of Newcastle Disease during the month in a province in china, and to establish some corresponding mathematical predicting models [13]. Soliman et al. have investigated the utility of deep learning with feedforward neural networks (DL with FNN) for Influenza like illness (ILI) prediction, in application to forecasting influenza in Dallas County based on meteorological data (Air Temperature, Relative Humidity(RH), Evapotranspiration (ET), Wind Speed, Solar radiation, Soil Temperature and Rainfall) [17]. The results obtained using the Deep Learning with Feedforward Neural Network were compared to the results obtained by other statistical models such as beta regression, Autoregressive Integrated Moving Average (ARIMA), Least Absolute Shrinkage and Selection Operators (LASSO), and non-parametric Multivariate Adaptive Regression Splines (MARS) models for one week and two weeks ahead forecasting. A probabilistic forecasting of influenza in Dallas County by fusing all the considered models using Bayesian model averaging (BMA) was also developed. Based on the results obtained, FNN and the BMA-based multi-model ensemble of ILI forecasts yield a similar competitive performance, outperforming all other considered models.

Mezzatesta et al. have performed a research on the prediction of province-level outbreaks of foot-and-mouth disease in Iran using a zero-inflated negative binomial model based on the number of previous occurrences of HFMD for the same or adjacent provinces and season as covariates [16]. Incidence prediction of communicable diseases has also been proposed by using Back Propagation Neural Network model based on population, earthquake intensity, route distance, direct distance, Normalized Difference Vegetation Index, Normalized Difference Water Index, and Digital Elevation Model [14]. A hybrid model using genetic algorithm and neural network for predicting dengue outbreak based on dengue and rainfall data has also been proposed [15].

#### 6.1.2 Classification problems for detecting disease outbreaks

On the other hand, most classification problems address the task of detecting disease outbreaks as shown in Table 6. For instance, mosquito-borne diseases include Chikungunya, Dengue fever, Yellow fever, Zika virus, and Lymphatic filariasis which is transmitted by Aedes aegypti mosquito. The female anopheles mosquito spreads Malaria and Lymphatic filariasis, whereas culex mosquito spreads Lymphatic filariasis and West Nile fever. Vijayakumar et al. incorporated personal information, diseases' signs/symptons and contextual information in building the Fog computing-based intelligent healthcare system for the detection and prevention of mosquito-borne diseases [49] The experimental evaluation revealed the best performance can be achieved using the Fuzzy k-Nearest Neighbour (FKNN) classifier with 95.9% classification accuracy. Khanita showed another approach that applies symptoms and location based method to detect disease epidemics using a symptom-based approach. *k*-NN clustering is also helpful to identify a new potential epidemic cluster [59]. SVC with RBF kernel and GridSearch algorithm were used in predicting the outbreak of cardiovascular diseases in Italy and America with accuracy of 95.25% based on 29 features that include Framingham risk factors, Uremic risk factors and inflammatory biomarkers [45]. Tapak et al. also investigated and compared the performance of three machine learning techniques of SVM, RF and ANN in detecting ILI outbreaks [22]. The total accuracy of the SVM (Gaussian Radial Basis (GRBF), polynomial, Sigmoid) was 89.2% which shows excellent performance.

Khanita showed that *k*-NN based classification method can be applied to detect disease epidemics using a symptom-based and location-based approach [59]. Vijayakumar et al. also have introduced a Fog computing-based intelligent healthcare system for the detection and prevention of mosquito-borne diseases [49] using a Fuzzy k-Nearest Neighbour (FKNN) classifier with 95.9% classification accuracy.

Chanlekha and Collier have also proposed a method that associates each reported event with the most specific spatial information available in a news report. This is useful not only for health surveillance systems, but also for other event-centered processing systems [47]. Based on the results obtained, the Conditional Random Fields (CRF), statistical machine learning was the approach that performed the best by yielding an F-score of 85.5% compared to probabilistic approach. There was an approach introduced for detecting disease outbreaks using fuzzy inference based on physiological variables: age, blood pressure, cholesterol, obesity, and smoking [43].

### 6.2 Types of datasets and parameters used

This section summarizes and discusses the findings in relation to the **RQ2**: What disease datasets in the literature have been used to build the models? and **RQ3**: What type of parameters or variables have been used?

Table 13 shows the type of diseases, dataset sources and related studies working on the prediction and detection in order to limit the spread of disease outbreaks. For instance, for *dengue* disease, most studies have used the *Epidemiology* and *Meteorological* data in order to perform the predictions and detections of dengue outbreaks.

**Table 13:** Diseases, Database Sources and Studies.

Diseases	Database Sources or Parameters
Dengue	Meteorological Data [15], [19], [20], [32], [34], [40], [41], [49]
	Epidemiology Data [26], [49], [52], [54]
	Demographic Data [32], [49]
	Social Media Data [34], [52]
	Remotely Sensed Data [20]
	Spatial Data [30]
Zika	Epidemiology Data [19], [49], [51], [58]
	Meteorological Data [49], [58]
	Demographic Data [49], [58]
HFMD	Meteorological [19], [42]
	Spatial Data [16]
	Search Keywords [44]
ILI	Social Media Data [21], [23], [29], [33], [37], [53], [68], [69], [70]
	Meteorological [17], [21], [23], [31], [33], [37], [68], [69]
	Search Keywords [21], [23], [33], [37], [68], [69]
	Epidemiology Data [22], [23], [24], [36], [59]
	Spatial Data [59]
Others	Epidemiology Data [13], [18], [27], [28], [35], [38], [39], [46], [50], [55], [57]
	Demographic Data [45], [50], [55], [57]
	Meteorological Data [25], [56]
	Spatial Data & Remotely Sensed Data [14]
	Social Media Data [46]
	News Articles [47]
	Search Keywords [48]

^⁎^Dependent variable: Number of disease outbreak incidences (EP1) (see Table 7).

For structured datasets, the most frequently used databases include the *Meteorological* and *Epidemiology* data. The temperature variable improves dengue outbreaks forecasts better than humidity and rainfall for the *Meteorological* data [40].

In contrast, for unstructured datasets, *Social Media* data and *Search Keyboards* are the most frequently used dataset for forecasting disease outbreaks (e.g., Influenza-like illness (ILI)). There are also studies conducted that used multiple sources of data such as Social Media Data, Search Keywords, Meteorological Data ([21], [23], [33], [37], [68], [69]) and also Epidemiology Data coupled with Demographic Data ([50], [55], [57]).

Incorporating multiple sources of data can be useful if there is a lack of data availability to predict and detect disease outbreaks [48]. For instance, the dynamics of certain diseases, (e.g., Dengue, Malaria, Zika) could be associated with other information (e.g., disease carriers density, population density and mobility), and this information should be incorporated in the process of modelling the spread of disease outbreaks and reduce the residual errors of the models [34]. There could also be a potential threat that may arise when the conducted analysis and the data used are dependent on a particular study of diseases. This threat can be handled by incorporating multiple data obtained from different studies [48]. For instance, incorporating epidemiological data that includes incidence, distribution, and control of diseases and meteorological data from different locations may produce more reliable results [50]. Besides that, several findings have also suggested that incorporating epidemiological, demography and meteorological data may also improve the performance of the forecasting algorithms [46], [48]. In addition to that, incorporating spatial information related to disease outbreak with the epidemiological data may also improve the epidemiological detection and prediction [47].

It also has been shown that incorporating data extracted from WSM with meteorological data, that is collected at a finer resolution, will also improve the performance of the disease detection system [19], [48].

Most unstructured data (e.g., blogs, news or social media medium) are not explored intensively. Corley et al. evaluated blog posts, a type of Web and Social Media (WSM), and they found that the number of blogs related to ILI has a high correlation with the number of ILI related reports done by patients during the outbreak of influenza season in US 2008–2009. In this work, the frequency of WSM posts was hypothesized to be highly correlated with the number of patient reporting ILI [70]. As a result, one may use the WSM to identify and extract information for predicting disease outbreak based on the sentiment characteristics and its location, and visualizing the obtained results using any data visualization tool. By incorporating the analysis of relevant information extracted from WSM, the spread of ILI diseases or any infectious diseases can be detected and predicted with more effectively and efficiently [29], [53].

In addition to that, not many works are done relating to methods examined and used to relate disease events that are officially reported in any news or reports to their exact GPS location and time of occurrences [47]. Words that have similar meanings can also be used to improve the relationship between reports and its spatial information. For instance, using effective methods for topic modelling (e.g., LDA), one can easily perform the topic modelling by identifying the main topic of each news article and cluster this news according to the topics. These topics or clusters information then could be used to improve the association between news articles and events [46], [50].

The detection of disease outbreak can also be improved by enhancing the preprocessing techniques for social media sources, such as extracting URLs information, removal of meaningless words (e.g., stop-words), reducing words into its root words (e.g., stemming), recognizing and extracting negative words and identifying and locating the GPS locations. The GPS location information is now embedded on the web social media sites and this information can be leveraged in future research for more advanced WSM surveillance system [53]. Most of the unstructured resources in the web social media, (e.g., twitter and facebook messages and blogs) can be effectively and efficiently examined and classified based on the Epidemiology related terms and its geo-location and the spread of infectious disease could be detected and predicted [53].

### 6.3 Type of problems addressed and individual machine learning models

This section summarizes and discusses the findings in relation to the **RQ4**: What type of problems are addressed using these machine learning models? and **RQ5**: What are the individual models (e.g., neural network, linear regression) used? which includes **RQ5.1**: What are the best performing individual models?

AI or machine learning methods can be categorized into supervised, unsupervised and semi-supervised learning. Supervised learning is the process of inferring a function from labeled training data which are used to handle the *classification* and *regression* problems in predicting and detecting the occurrence of disease outbreaks efficiently and effectively. These algorithms include Support Vector Machine (SVM), Decision Tree, Random Forest, Naïve Bayes (NB), Artificial Neural Network (ANN), Bootstrap Aggregating, AdaBoost,

In contrast, unsupervised learning methods can be used for *clustering* and *dimensionality reduction* problems. For instance, Principal Component Analysis (PCA) can be used to transform a data into another dimension with reduced number of features, which would improve the learning process [71]. Other unsupervised learning methods, such as *k*-means clustering, can be used to describe the data by clustering the data into smaller groups or subgroups and also can be used to detect outliers. In addition to that, for unstructured data, topic modelling algorithms or methods (e.g., Latent Dirichlet allocation (LDA)) could be used to identify relevant topics from infectious disease textual record [47], [48].

#### 6.3.1 Approaches to solving regression problems

The approaches to solving regression problems in detecting and predicting the occurrence of disease outbreaks can be divided into statistical and machine learning approaches.

Based on the information tabulated in Table 9, for the statistical approaches, several models have been used to perform the detection and prediction of disease outbreaks that includes ARMA [13], ARIMA [17], [22], [23], [25], [27], [38], [39], [42], SARIMA [25], [26], [40], [41] and LASSO [17], [19]. In time series modeling, CNN has outperformed the nonlinear autoregressive exogenous model (NARX) [29]. Based on the review, deep learning algorithms have outperformed the statistical approaches in detecting and predicting the outbreaks of disease, such as ARIMA [23], [25], [33], [42] and SARIMA [25].

In machine learning approach, most of the best methods found to be more effective in predicting disease outbreaks are those related to neural network family. The experimental results showed the consistent performance improvements by the proposed deep learning approaches over other representative linear and non-linear methods on multiple real-world datasets. These algorithms include the Long Short Term Memory (LSTM) [17], [23], [25], [31], [32], [33], Convolutinal Neural Network (CNN) [29], Back Propagation Neural Network (BPNN) [14], [42], Multilayer Perceptron (MLP) [20], Neural Network [22], Hybrid Neural Network (HNN) [15] and combination of statistic and deep learning approaches [38], [39].

LSTM (RNN), was also able to produce better predictive capability for predicting the morbidity incidence of 10 infectious diseases, compared to linear model (RR), time series analysis model (ARIMA), boosting tree model (XGBoost) [23]. LSTM algorithms was shown to be more superior in predicting Malaria outbreak [25] with RMSE of 0.072.

Based on the results, a hybrid approach is also found to be more effective in predicting disease outbreaks. For instance, a hybrid method that combines Autoregressive Integrated Moving Average (ARIMA) and Generalized Regression Neural Network (GRNN) has shown better performances compared to single individual models in forecasting hepatitis incidence in Heng County, China [38] and predicting haemorrhagic fever with renal syndrome in China [39]. The results also showed that the data fitting were good for the proposed hybrid approaches [38], [39] although the results showed that better performance can be obtained for short term prediction [40], [41].

The Random Forest algorithm produces good performance but BPNN is a better algorithm [42], [51]. For instance, the Random Forest (homogenous ensemble learning) approach produced better results compared to the ARIMA approach in predicting the H5N1 avian outbreaks in birds in Egypt [27].

For other algorithms, Tapak et al. have investigated and compared the performance of four machine learning techniques of SVM, ARIMA, RF and ANN in forecasting weekly number of influenza-like illness (ILI) cases with time series adaptation of them [22]. Based on the results obtained, the sensitivity of the ANN for the test set (86.2%) was better compared to the other three methods. In addition to that, Scavuzzo et al. have made a performance comparison between 6 models which comprises of two linear models (Simple and Ridge) and four non-linear models (Support Vector Machine, ANN multi-layer Perceptron, Decision Tree, and K-Nearest Neighbor) [20]. The modelling was conducted based on several variables that include Normalized Difference Vegetation Index (NDVI), Normalized Difference Water Index (NDWI), Land Surface Temperature (LST) night, Land Surface Temperature (LST) day and TRMM-GPM rain (vegetation, moisture, temperature and rain). The ANN multilayer perceptron (MLP) is found to be the model that can best produce more presentable results compared to other models [20].

A feature selection based Time Series forecasting has been proposed for predicting future outbreaks of Methicilin-resistant Staphylococcus aereus (MRSA) [18]. The performance of the feature selection methods has been measured using the root mean square error (RMSE) and mean absolute error (MAE) performance metrics with RMSE and MAR values of 0.1349 and 0.1003 respectively. The six regression algorithms Gaussian Processes(GP), Support Vector Machine (SVM), *k* Nearest Neighbour (*k*NN), Random Forest (RF), Linear Regression (LR), Multilayer Perceptron (MLP) have been applied in this work and the best results are obtained using the GP and SVM methods. The work proposed a multi-objective evolutionary algorithm to find the best regression algorithm (ensemble learning) at prediction intervals.

#### 6.3.2 Approaches to solving classification problems

Based on the information tabulated in Table 10, Neural Network methods were also found to be very effective also in detecting disease outbreak. This review reports that the neural network based methods have achieved 4 best results out of 14 studies [44], [46], [51], [55].

SENTINEL, a deep learning based algorithm has been proposed to classify health-related tweets with high accuracy classification results. In this work, it has shown that deep neural network algorithms (e.g., CNN and LSTM) have outperformed the Multinomial Naïve Bayes (F_1_ of 0.852 for Twitter classification) and SVM models (F1 of 0.939 for news classification) [46]. In a separate work, multiple machine learning method were also used to predict the amount and time of the outbreak of HFMD and these methods include Ridge Regression, KNN, RF and RNN algorithms, having the best AUC of 0.9164 for validation set and 0.8413 for testing set using the LSTM model [44], in which a transfer learning (TL) was used in training the LSTM in order to improve the generalization ability of the LSTM model [32]. This indicates that deep learning algorithms (e.g., LSTM (RNN)) performed better than any classical linear model statistical machine learning (e.g., classical linear model (ridge regression), statistical machine learning (K-nearest neighbor), and homogenous ensemble learning method (random forest)) [32], [44].

Jiang et al. compared the performance of three machine learning (e.g., BPNN, GBM and RF) in predicting the occurrence of Zika based on five factors; Occurrence of Aedes, Absence records, predicted distribution of Aedes, Meteorological factors, Environment factors, socioeconomic factors. The BPNN model obtained the best result having the area under the curve (AUC) of 0.966 [51]. In predicting the meningitis outbreaks in Nigeria, several machine learning methods, namely, logistic regression, k-nearest neighbors (KNNs), random forests, support vector machine (SVMs) and neural networks (NNs) were applied and their accuracy of prediction were compared in which the neural network algorithm achieved an accuracy of over 95% [55].

*k*-Nearest Neighbour (*k*-NN) was found to be very effective when applied for infection detection using physiological and social data in social environments [48], detection and prevention of mosquito-borne diseases [49] and detection of disease epidemics using a symptom-based approach [59]. In short, Neural Network and *k*-Nearest Neighbor methods were found to be popular and very effective in detecting and predicting disease outbreaks [48].

### 6.4 Assessment measures and methods

This section summarizes and discusses the findings in relation to the **RQ6**: What are the evaluation measures used to assess the performance (e.g., Accuracy, Precision, Recall, F-Measure, ROC) of the proposed machine learning algorithms (e.g., prediction models, detection models, classification models)?

In most regression problems, all the proposed methods or algorithms are measured by using MAE, MSE, RMSE and MAPE. On the other hand, Accuracy and ROC are mostly used for evaluating the performance of the classifiers proposed in those studies. In this paper, 17 out of 34 (50%) studies found that the individual models that belong to neural network family performed better when compared to other linear and non-linear methods.

Table 9, Table 10 show that machine learning models achieved lower MAE and MSE measurements compared to other statistical models (e.g., ARMA (0,1) and SARIMA) [15], [22]. Similarly, it can be observed from these tables that deep learning approaches produced lower RMSE readings [25], [31]. As we have noticed based on summaries stated in previous sections that machine learning approaches performed better than the statistical approach. For the MAPE measurement, there is an inconsistent trend shown above. Deep learning algorithms are found to show consistent trend in producing higher accuracy measurements [49], [55], $F_{1}$ Score measurement [46] and ROC measurement [51] compared to other statistical and machine learning models reviewed in this study.

### 6.5 Ensemble methods

This section summarizes and discusses the findings in relation to the **RQ7**: What ensemble models (e.g., stacking, bagging, boosting) are used?

There are several ensemble approaches introduced to forecast disease outbreaks. Table 11, Table 12 outline several ensemble approaches used to predict and detect disease outcomes and also summarize the evaluation approaches and measures used for ensemble learning. There is a need to explore further the capability of ensemble models or hybrid models based on deep learning methods using multi-source data, as these have been shown to improve the performance of the base model.

An ensemble method can be defined as a technique which uses multiple independent similar or different models/weak learners in order to derive an output. Ensemble methods can be categorized into bagging, boosting and stacking approaches.

Bagging is a homogeneous weak learners' model that are arranged independently in parallel and combines their outputs or prediction for determining the final output. For instance, a novel bagging type of ensemble model developed and called Ensemble Penalized Regression Algorithm (EPRA) has outperformed other individual models (e.g., LASSO, Ridge, Elastic Net, SCAD and MCP) for timely tracking the timing and magnitude of dengue epidemics based on multi-sources data (e.g., search keywords Data, meteorological data and social media data) by integrating different penalties with the techniques of iteratively sampling and model averaging [34]. These findings can be used as indications or trends that can be monitored online informally to estimate and detect the temporal patterns of disease epidemics in other parts of the world. Rider and Chawla developed an approach that allows the sharing of beneficial information while staying within the bounds of data privacy. Three bagging types of ensembles have been proposed called ensemble Dirichlet Process Mixture Model (DPMM), ensemble COOC (DPMM trained on disease occurrence) and ensemble Co-occurrence Based Clustering (CBC) [57]. Based on the results obtained, ensemble approaches produced better accuracy performance. Another example of bagging approach named bootstrap-aggregated ensemble of fine decision trees, to identify epidemic risk areas has also been proposed [58]. This approach has shown to be capable to infer about possible epidemic risk areas caused by the ZIKA virus, which can lead to severe complications for pregnancy.

On the other hand, boosting approach has homogeneous weak learners that are arranged sequentially and adaptively to improve model predictions of a learning algorithm. For instance, a statistical based algorithm coupled with ensemble algorithm, in which a SARIMA model and XGBoost model are combined using a mechanism that allows a self-adaptive weight adjustment, produced better results compared to LASSO and LSTM alone [21]. The same ensemble has been proven to be more effective compared XGBoost alone [37]. A hybrid method has also been proposed that combines CNN, RNN and residual links to produce ensemble model which is more expressive and is able to perform a more robust prediction of epidemiological data. Based on the results obtained, it showed that this hybrid method outperformed AR, VAR and GPS algorithms with RMSE of 0.259 [24].

Another approach of ensemble learning is called stacking approach that often considers heterogeneous weak learners, learns them in parallel and combines them by training a meta-model to output a prediction based on the different weak models predictions. Stacking algorithms are shown to be more superior in predicting Malaria outbreak [25] with RMSE of 0.068. The stacking ensemble method comprises of four machine learning algorithms (ARIMA, SARIMA, BPNN, LSTM). Similarly, a stacking ensemble approach has been proposed where a fusion method called RKRE based on both ResNet and KG_ResNet in which the expert system attained an average classification accuracy of 88.57%, which is a good feasibility study in the field of disease classification [50]. In this study, a combination of knowledge graph (KG) and Deep Learning algorithms (ResNet + KG_ResNet (RKRE)) was introduced to classify diseases in order to detect the disease outbreaks.

Another common type of ensemble used is a Bayes optimal classifier. For instance, an ensemble approach call superensemble was proposed that combines Bayesian Weighted Outbreaks (BWO), a process-based model (SIR-EAKF) that combines ensemble adjusted Kalman filter (EAKF) with a dynamical Susceptible-Infected-Recovered (SIR) mode, and a simple null model [36]. In this ensemble approach, all three models are integrated to produce a single model classifier and the performance is better compared to individual models. In a separate work, Chowel et al. generated a sequential short-term based forecast system for epidemic outbreaks by combining the Generalized-Growth Model (GGM) and the Generalized Logistic Model (GLM). The GGM-GLM ensemble model produced an overall mean RMSE performance of 0.374 in the Ebola Forecasting Challenge [35].

Based on this review, several bagging, stacking and boosting approaches have been identified and bagging approach was found to be more popular and produced better performance results compared to individual model approach.

## 7 Conclusion

In conclusion, the aim of this literature review is to identify and analyse various approaches, types of datasets, types of parameters or variables, individual models, ensemble models, performance measures and approaches used in the previous works on leveraging machine learning approaches to limit the spread of deadly disease outbreaks. In this work, there were six online digital libraries used to retrieve all related peer-reviewed articles and only forty-seven studies have been selected between the year of 2010 and 2020 publications in which seven main questions are used to assess the quality of these studies. This SLR was conducted to evaluate and select all relevant research studies related to the detection and prediction of disease outbreaks using machine learning based on the seven questions outlined earlier.

The contributions of this paper can be summarized as follows:

• The type of databases and variables used are identified, and Meteorological and Epidemiology data are found to be the mostly useful datasets for predicting and detecting disease outbreaks.
• Multi-sources data contributes to the improvement of the disease outbreaks predictions.
• Algorithms belong to the Neural Network family are found to provide better performance compared to other linear and non-linear machine learning methods.
• Ensemble and hybrid approaches performed better and are more appropriate to be applied for predicting and detecting disease outbreaks.
• Exploring unstructured data (e.g., news, blogs, search keyword trends) may improve the performance of the disease outbreaks prediction and detection.

Several guidelines are generated based on the findings obtained from this SLR for future work. Firstly, there is a need to explore further the capability of ensemble models or hybrid models based on deep learning methods using multi-source data, as these have been shown to improve the performance of the base model. Next, A limited number of investigations conducted in the area of disease outbreaks prediction based on multi-sources data as the findings from existing studies have shown that a more comprehensive understanding can be obtained about a particular disease outbreak by integrating multi-sources data. We can produce better modelling results comprehensively by analysing these complex relationships among multi-sources data. Finally, limited works are found in exploring unstructured data such as news articles, blogs and web social media sites, even though integrating structured and unstructured data, has been shown to improve the prediction of disease outbreaks.

## Declarations

### Author contribution statement

All authors listed have significantly contributed to the development and the writing of this article.

### Funding statement

This work was supported by the Universiti Malaysia Sabah internal grant no. SBK0302-TK-2016, SBK0428-2018 and SDN0076-2019.

### Data availability statement

No data was used for the research described in the article.

### Declaration of interests statement

The authors declare no conflict of interest.

### Additional information

No additional information is available for this paper.
